# Supplementary material for: Predictive models for short-term load forecasting in the UK’s electrical grid
Source: PLoS One. 2024 Apr 4;19(4):e0297267. doi: 10.1371/journal.pone.0297267 (PMC10994373; doi:10.1371/journal.pone.0297267)
Supplement: Attachment — Submitted filename: review comments PONE-D-23-39140_reviewer.pdf [file pone.0297267.s001.pdf]

TITLE: Predictive models for short-term load forecasting in the UK's electrical grid

Manuscript Number : PONE-D-23-39140_reviewer

**Type of manuscript : ** Original Article

Summary
In this paper, the authors used machine learning models for short-term load forecasting in Great Britain's power system using a unique data set incorporating generation, conventional, renewable, and interconnectors to approximate total electricity demand.
I found this paper interesting and important in the energy sector. However, the manuscript requires some improvements. Some general comments are:

General Comments

- In the Abstract, the authors should briefly present and justify the novelty of their approach in contrast to the existing body of knowledge.
- Provide a list of abbreviations used in the paper.
- What is the justification of the evaluation metrics used in this study, RMSE, MAD, MAPE and R? It is also usually recommended to use MASE when modelling time series data. How did you select the best kernel for each SVR and GPR?
- Include a separate "Discussion" section. In this section, the authors should present their findings and their main implications, highlight their study's limitations, and briefly mention some precise directions they intend to follow in their future research work. Can the authors mention how much of their research is influenced by the data they used or to which extent the methodology used within the developed research can be easily applied to other situations when the datasets differ? In this way, the authors could highlight their approach's generalisation capability to justify a wider contribution to the current state of the art.
- In the "Conclusion" section, authors should avoid summarising the aspects they have already stated in the body of the manuscript. Instead, they should interpret their findings at a higher level of abstraction than in the previous sections of the manuscript. The authors should highlight whether or to what extent they have addressed the necessity identified within the "Introduction" section (the identified gap). The authors should avoid restating everything they did once again. However, instead, they should emphasise what their findings mean to the readers, making the "Conclusions" section interesting and memorable to them. The authors should not restate what they have done or what the article does. They should focus instead on what they have discovered and, most importantly, on what their findings mean.
