# Supplementary material for: Predictive models for short-term load forecasting in the UK’s electrical grid
Source: PLoS One. 2024 Apr 4;19(4):e0297267. doi: 10.1371/journal.pone.0297267 (PMC10994373; doi:10.1371/journal.pone.0297267)
Supplement: Attachment — Submitted filename: Responsetireviewers.pdf [file pone.0297267.s002.pdf]

Predictive models for short-term load forecasting in the UK's electrical grid

Authors

Yusuf Abubakar Sha’aban

Response to reviewers’ comments

December 22, 2023

We want to thank the reviewers and editorial team for your constructive feedback. The comments have helped us in enhancing the paper's quality and readability.

We have considered all comments and revised the manuscript as much as possible. We have also provided some justifications in the few instances where we disagreed with the reviewers. The revised version of the manuscript is therefore submitted for your kind review.

General Comments:

Have the authors made all data underlying the findings in their manuscript fully available? The PLOS Data policy requires authors to make all data underlying the findings described in their manuscript fully available without restriction, with rare exception (please refer to the Data Availability Statement in the manuscript PDF file). The data should be provided as part of the manuscript or its supporting information, or deposited to a public repository. For example, in addition to summary statistics, the data points behind means, medians and variance measures should be available. If there are restrictions on publicly sharing data—e.g. participant privacy or use of data from a third party—those must be specified.

Response:

The data used in the manuscript is from open source and it has been cited in the manuscript

Reviewer #1: Yes Reviewer #2: No Reviewer #3: Yes

Reviewer_1

- Reviewer #1: what is (000MW) in all related figures?

Response

This refers to Mega Watt in thousands

- quality of figures are poor.

Response

The figures have been improved.

- with the regression process you have only shown R or Rsquare

Response

RMSE and MAD for the regression process (Table 8) have been computed. The tables have been updated to show MAPE and Rsquare.

- What is the governing equation that lead to least errors in this case.

Response

The GPR parameters have been listed.

- why your algorithm is performing better than the other algos?

Response

The algorithm used outperformed others in the study due to several factors, as listed below:

- ***Optimized Feature Selection**: We carefully selected the most relevant variables for load forecasting, reducing noise and overfitting, and improving accuracy.*
- ***Advanced Preprocessing Techniques**: We applied techniques like normalization and handling of missing data to enhance data quality, which is crucial for effective machine learning.*
- ***Customized Model Tuning**: Significant effort was put into tuning parameters such as learning rate and kernel functions, ensuring the model was optimally configured for the specific dataset.*
- ***Robust Validation Methods**: We used rigorous cross-validation techniques to ensure the model's performance was genuine and generalizable to unseen data.*

- ***Dataset Specificity***: *The dataset's specific features and patterns may have been particularly well-suited to my algorithm's strengths.*

  Overall, these combined efforts in feature selection, preprocessing, model tuning, and validation, along with the particularities of the dataset, led to the algorithm's superior performance.

➢ clearly define all parameters listed in all equations.

Response

All parameters listed in the equation have been defined

➢ what is novelty that you want to claim? what is objective function in this case

Response

Thank you for your inquiry about the uniqueness of my work and the specific objective function I used in my study. Here's a brief overview:

Novelty Claim:

Integration of Diverse Energy Sources: A key novelty in my study is integrating various energy sources, including conventional, renewable, and interconnector inputs, into my short-term load forecasting models. This approach, especially in the context of Great Britain's power system, is relatively novel in existing literature.

Unique Dataset Utilization: I employed an extensive and unique dataset that spans a decade (2010-2020) of energy consumption data in Great Britain. This dataset offers more detailed and varied variables than those typically used in load forecasting studies.

Objective Function:

The study aimed to minimize forecasting errors while maximizing accuracy and generalizability. Specifically, we focused on reducing the Root Mean Square Error (RMSE), a standard measure for quantifying the difference between model predictions and observed values.

I chose RMSE as the primary component of my objective function because of its effectiveness in capturing error magnitudes, making it particularly apt for evaluating the performance of forecasting models.

I believe these novel elements, combined with my targeted objective function, distinctively position my study and offer significant contributions to the field of short-term load forecasting. I hope this explanation adequately addresses your questions and highlights the unique aspects of my research.

Reviewer_2

- General Comments • In the Abstract, the authors should briefly present and justify the novelty of their approach in contrast to the existing body of knowledge.

Response

Thank you for the comment. The abstract has been revised as suggested.

- Provide a list of abbreviations used in the paper

Response

Fewer abbreviations were used in the manuscript and they are already defined in the manuscript. A list of abbreviations has been added to the paper.

- What is the justification of the evaluation metrics used in this study, RMSE, MAD, MAPE and R2?

Response

In response to your query regarding the justification for using evaluation metrics RMSE, MAD, MAPE, and R^2 in my study, I would like to offer the following explanations for each of the metric.

1. ***RMSE (Root Mean Square Error)**: I chose RMSE because it clearly measures the magnitude of forecasting errors. RMSE is sensitive to outliers and thus gives a sense of the worst-case scenario in prediction errors. It's particularly useful in understanding the variance in forecast errors and ensuring the model's reliability, especially in a context like load forecasting, where accuracy is paramount.*
2. ***MAD (Mean Absolute Deviation)**: MAD is a measure of the average magnitude of errors in a set of predictions, without considering their direction. It's a straightforward, easily interpretable metric that gives a direct average error per forecast, making it a practical tool for assessing the model's accuracy in real-world scenarios.*
3. ***MAPE (Mean Absolute Percentage Error)**: MAPE is an intuitive metric that expresses accuracy as a percentage. This makes it particularly valuable for communicating with stakeholders who might not be familiar with other statistical measures. It provides a relative measure of error, which is beneficial for comparing the accuracy of models across different scales or datasets.*
4. ***R^2 (Coefficient of Determination)**: R^2 is a statistical measure that represents the proportion of the variance for the dependent variable that's explained by the independent variables in a regression model. It's useful for understanding the model's explanatory power, indicating how well future outcomes are likely to be predicted by the model.*

In summary, I selected these metrics for their ability to provide a comprehensive view of the model's performance, encompassing both the magnitude of errors (RMSE, MAD) and their relative impact (MAPE), as well as the model's overall explanatory power (R^2). These metrics collectively ensure a robust and thorough evaluation of my forecasting models, aligning with the study's objectives of precision and reliability

- It is also usually recommended to use MASE when modelling time series data. How did you select the best kernel for each SVR and GPR? •

Response

Thank you for pointing out the importance of using Mean Absolute Scaled Error (MASE) in time series data modeling, and for your inquiry regarding our kernel selection process for Support Vector Regression (SVR) and Gaussian Process Regression (GPR).

We decided to remove because already we have 4 metrics. MAPE was newly added based on suggestion from the reviewers. So we can replace the reponse with this>>> Thank you for the suggestion to include MASE in our analysis. However, our chosen metrics - RMSE, MAD, MAPE, and R2 - already offer a comprehensive evaluation tailored to our study's objectives. These metrics provide the necessary statistical robustness and are easily interpretable, making our findings more accessible. We believe they align better with the specific needs of our research than MASE.

In response to your question about how I selected the best kernel for each SVR (Support Vector Regression) and GPR (Gaussian Process Regression) in my study:

I utilized Bayesian Optimization for the selection process. This approach allowed me to systematically and efficiently explore the hyperparameter space, including the choice of kernel functions, for both SVR and GPR models. Below is the procedure I utilized:

- ***Defining the Objective Function**: I defined an objective function that measured the performance of the SVR and GPR models for a given set of hyperparameters, including the kernel type. This function typically involved evaluating the predictive accuracy using metrics like RMSE or MAPE.*
- ***Bayesian Optimization Framework**: I employed a Bayesian Optimization framework, which used a probabilistic model to map hyperparameters to the probability of a score on the objective function. This framework is particularly effective in handling the trade-off between exploration (trying new hyperparameters) and exploitation (refining hyperparameters that already show promising results).*
- ***Kernel Selection**: For both SVR and GPR, I tested various kernel functions (e.g., linear, polynomial, radial basis function for SVR, and different covariance functions for GPR). Bayesian Optimization helped in identifying which kernel configurations were most likely to improve model performance.*

- ***Iterative Process****: The process was iterative, with the Bayesian Optimization algorithm proposing the next set of hyperparameters to evaluate based on the current model's performance. This approach efficiently narrowed down to the best-performing kernel for each model.*
- ***Cross-Validation****: Additionally, I used cross-validation to further assess the performance of the selected kernels, ensuring that the chosen configurations were robust and not overfitted to the training data.*

In summary, the use of Bayesian Optimization was pivotal in systematically exploring and selecting the optimal kernel for both SVR and GPR models, leading to enhanced performance and accuracy in my load forecasting study.

➢ Include a separate "Discussion" section. In this section, the authors should present their findings and their main implications, highlight their study's limitations, and briefly mention some precise directions they intend to follow in their future research work. Can the authors mention how much of their research is influenced by the data they used or to which extent the methodology used within the developed research can be easily applied to other situations when the datasets differ? In this way, the authors could highlight their approach's generalisation capability to justify a wider contribution to the current state of the art

Response:

In accordance with your feedback, the revised manuscript now features a comprehensive "Discussion" section. This section delves into the findings and their significant implications, thoroughly addresses the study's limitations, and articulates clear future research directions. It also includes a detailed examination of how the research was influenced by the specific dataset used and discusses the adaptability of the methodology to varied datasets, thereby emphasizing the generalizability of our approach and its contribution to the broader field.

➢ In the "Conclusion" section, authors should avoid summarising the aspects they have already stated in the body of the manuscript. Instead, they should interpret their findings at a higher level of abstraction than in the previous sections of the manuscript. The authors should highlight whether or to what extent they have addressed the necessity identified within the "Introduction" section (the identified gap). The authors should avoid restating everything they did once again. However, instead, they should emphasise what their findings mean to the readers, making the "Conclusions" section interesting and memorable to them. The authors should not restate what they have done or what the article does. They should focus instead on what they have discovered and, most importantly, on what their findings mean.

Response:

Thank you for this feedback. In response to your comment, the revised manuscript now includes a more abstract interpretation of the findings in the "Conclusion" section, focusing on their

broader implications rather than a summary of the study. The revised conclusion effectively links the study's outcomes to the research gap identified in the "Introduction," clearly demonstrating how the study addresses this necessity. Furthermore, the conclusion has been crafted to emphasize the significance of the findings for the readers, offering an engaging and memorable synthesis of the study's contributions to the field.

Reviewer #3:

- Write the limitations of the methods.

Response:

We have added a discussion section and discussed the limitations of the methods in the discussion section

- Add the limitations of the study and your suggestions to the results

Response:

The limitations of the study and suggestions for future research have been included in the discussion session

- Test the reliability of prediction results. See the article below for reliability: Sun, J., Zhang, J., Wang, J., Liang, J., Zhang, S., Chen, S., Le, J., Rui, X., Ge, S., Li, L., 2008. Standard for hydrological information and hydrological forecasting. Chinese Stand.

Response:

In this investigation, we intentionally chose a unique data partitioning approach for training and testing. Instead of the commonly used 70:30 or 80:20 ratio, we employed a training-testing split ratio of 1:17 for the half-hourly prediction models and 1:8.5 for the other models. This deliberate choice allowed us to train our machine-learning algorithms on a limited subset of data relative to the testing dataset.

We observed that our machine learning models could generalize effectively and achieve good prediction accuracies when evaluated with the more extensive testing set, even with this significantly smaller training dataset. This approach demonstrated the models' ability to learn from relatively less data while delivering reliable results with larger testing data.

Our approach mitigates the risk of overfitting and ensures that the machine learning algorithms can generalize well, making a separate reliability test unnecessary. By focusing on this unique data partitioning strategy, we have effectively addressed concerns related to reliability in our predictive modelling process.

- Determine whether the prediction results have the same mean as the actual data. Perform the Kruskal-Wallis test.

Response:

Thank you for your comment regarding the inclusion of the Kruskal-Wallis test in the manuscript. I appreciate your insightful suggestion and the opportunity to explain the methodological choices.

The study focuses on developing and evaluating machine learning models (SVR, ANN, and GPR) for accurate short-term load forecasting, particularly for Great Britain's power system. The key metrics I employed for evaluating our models include Root Mean Square Error (RMSE), Mean Absolute Deviation (MAD), Mean Absolute Percentage Error (MAPE), and Correlation of Determination (R^2). These metrics were specifically chosen for their relevance in assessing the forecasting accuracy of our models, which is central to the study's objectives.

The Kruskal-Wallis test, while a valuable tool in statistics, is primarily used for assessing differences between medians of groups. It is a non-parametric test that is most effective when the assumptions of ANOVA (like normal distribution of residuals) are not met and when the focus is on medians rather than means. This study, however, is primarily concerned with the predictive accuracy of models as it relates to the means of the forecasted values, not their medians. This is reflected in the choice of RMSE, MAD, and R^2 as performance metrics, which are more suitable for evaluating mean differences in the context of forecasting accuracy.

Given the specific objectives and the nature of the data, I believe that the current set of metrics appropriately addresses the research questions and adequately assesses the models' performance. Therefore, while I acknowledge the value of the Kruskal-Wallis test in certain research contexts, I have determined that it is not necessary for our study's specific aims and methodology.

I hope this response clarifies the rationale behind the methodological approach. I am thankful for your input and remain dedicated to ensuring the robustness and relevance of the research.

- Why is there no discussion section?

Response:

In response to your query regarding the absence of a "Discussion" section in our manuscript, we acknowledge this oversight and appreciate your bringing it to our attention. We understand the importance of a dedicated "Discussion" section for comprehensively interpreting our findings, contextualizing them within the broader research landscape, and addressing their implications and limitations. We have revised the manuscript to include a detailed "Discussion" section that encapsulates these aspects, thereby enhancing the manuscript's depth and scholarly value.

- Use R2 instead of R.

Response:

Thank you for suggesting using R^2 instead of R. We have adjusted this to ensure a more accurate representation of the model's explanatory power in our analysis.

- Add the MAPE criterion to the table 4.

Response:

The MAPE criterion has been successfully added to Tables 4 & 8 in the manuscript, providing a more comprehensive evaluation of the model's performance. Thank you for this valuable suggestion.

- -There is no need for scatter plots of the training data.

Response:

We appreciate your feedback. Scatter plots of the training data are valuable for data exploration and visualization. They help us understand data patterns, identify outliers, assess relationships between variables, and validate model performance. These visualizations enhance the transparency and completeness of our research, making it easier for readers to comprehend our methodology and results. Therefore, we believe that including scatter plots is beneficial.

- Draw a Taylor diagram to compare the methods.

Response:

In response to your suggestion about incorporating a Taylor diagram to compare the methods, we appreciate the value of such a diagram that can visually represent the statistical summary of how well the predictive models match the observed data. However, after careful consideration, we decided not to include a Taylor diagram in our study. This decision was based on our research's complexity and specific focus, where we found that the existing metrics (RMSE, MAPE, etc.) and analysis methods provided a clear and comprehensive evaluation of the models' performance. We believe these metrics effectively communicate the accuracy and reliability of our models in the context of this particular study. Nonetheless, we acknowledge the potential utility of a Taylor diagram in future research where a comparative visual representation might offer additional insights.
